# Taylor spatial frame in the treatment of open tibial shaft fractures

**DOI:** 10.4103/0019-5413.43393

**Published:** 2008

**Authors:** Mohammed J Al-Sayyad

**Affiliations:** Department of Orthopedic Surgery, King Abdulaziz University Hospital, Jeddah, Saudi Arabia

**Keywords:** External fixation, Taylor spatial frame, open tibial fracture

## Abstract

**Background:**

The Taylor spatial frame (TSF) is a modern multiplanar external fixator that combines the ease of application and computer accuracy in the reduction of fractures. A retrospective review of our prospective TSF database for the use of this device for treating open tibial fractures in pediatric, adolescent, and adult patients was carried out to determine the effectiveness and complications of TSF in the treatment of these fractures.

**Materials and Methods:**

Nineteen male patients with open tibial fractures were included. Of these fractures, 10 were Gustilo Type II, five were Gustilo Type IIIA (two had delayed primary closure and three had split thickness skin grafting), and four were Gustilo Type IIIB (all had rotational flaps). Twelve of our patients presented immediately to the emergency room, and the remaining seven cases presented at a mean of 3 months (range, 2.2-4.5 months) after the initial injury. The fractures were located in proximal third (n=1), proximal/middle junction (n=2), middle third (n=3), middle/distal junction (n=8), distal third (n=3), and segmental fractures (n=2). Patients were of an average age of 26 years (range, 6-45years). Mean duration of follow-up was 3.5 years.

**Results:**

All fractures healed over a mean of 25 weeks (range, 9-46 weeks). All were able to participate in the activities of daily living without any difficulty and most were involved in sports during the last follow-up. Postoperative complications included pin tract infection in 12 patients.

**Conclusion:**

The TSF is an effective definitive method of open tibial fracture care with the advantage of early mobilization, ease of soft tissue management through gradual fracture reduction, and the ability to postoperatively manipulate the fracture into excellent alignment.

## INTRODUCTION

Management of open tibial fractures continues to be a major therapeutic problem. Preventing infection, obtaining union, and returning the involved limb to normal function often remain elusive goals. Aggressive soft tissue management in conjunction with external fixation has given satisfactory results.^1^–^4^

Historically, external fixators have been reserved for the more severe, high-energy fractures with attendant soft tissue damage and comminution. In the orthopedic literature, external fixation generally fairs more poorly than other operative means such as open reduction with internal fixation or intramedullary nails. External fixation has been associated with a higher incidence of loss of reduction, delayed time to fracture healing, malunion, nonunion, and the complaint of frequent pin site infections.^1^,^5^ These findings are related to the use of uniplanar types of fixators, which are virtually impossible to adjust postoperatively. These types of fixators are also biomechanically inferior when compared to the multiplanar circular fixators popularized by Ilizarov, which provide better resistance to bending and torsion. The difficulty in application is the common complaint about the Ilizarov fixator for fracture care. In addition, the methods of fracture reduction with the use of olive and arch wires were difficult to master.^5^ The Ilizarov method has been used successfully in the treatment of tibial fractures, nonunions, and malunions, deformity, and shortening.^6^–^11^ The dynamic frame enables gradual lengthening, deformity correction, and nonunion or delayed union compression while remaining minimally invasive.^12^–^16^

The Taylor spatial frame (TSF) (Smith and Nephew Richards, Memphis, TN) has solved most of the previously mentioned complaints specifically the ease of frame application, technically easy in obtaining fracture reduction without the need to use complex Ilizarov fracture reduction techniques, and being able to easily adjust the frame postoperatively. A device is now available that can be applied easily and rapidly to a fractured limb. It provides all the advantages of multiplanar fixation of the Ilizarov system and even exceeds it in stability.^5^ In addition, accurate fracture reduction is now possible and easy to perform with the aid of computer accuracy.^5^

The purpose of this article is to determine the effectiveness and complications of the use of TSF in the treatment of open tibial shaft fractures.

## MATERIALS AND METHODS

All patients with open tibial shaft fracture who were admitted or referred to our facility and consented to external fixation treatment were included in the study, as this is the author's choice of treatment for such a fracture.

From February 2001 through February 2006, 19 open tibial shaft fracture were identified from our prospective TSF database. A retrospective review of these 19 patients was carried out. All patients were followed until after fracture healing. No patients were lost to follow-up. Patient charts were retrospectively reviewed for demographic data, mechanism of injury, associated injuries, surgical data, complications, number of operative procedures after the index admission procedure (formal surgical procedures performed in the operating room after the index admission procedure, i.e., procedures for subsequent irrigation and debridement, delayed primary closure, soft tissue coverage, bone grafting were also included), and functional outcome. Radiographs were reviewed to asses fracture type, location, displacement, angulation, fracture union, and final postoperative alignment.

All patients were males. The average age of the patients at the time of injury was 26 years (range, 6-45 years). Mean follow-up was 3.5 years (range, 2-5 years). The mechanism of injury was a motor vehicle accident in 13 patients, car vs pedestrian in four patients, and all-terrain vehicle injury in two patients. Table 1 provides a summary of patients' details.

Twelve of our patients presented through the emergency room immediately after the injury, and the remaining seven cases presented mean of 3 months (range, 2.2-4.5 months) after the initial injury. Fracture location was based on subdivision of the tibia into five regions: proximal third, proximal/middle junction, middle third, middle/distal junction, and distal third. A sixth category “segmental” was used for fractures involving two noncontiguous regions.^17^ Fracture location was proximal third (n=1), proximal/middle junction (n=2), middle third (n=3), middle/distal junction (n=8), distal third (n=3), and segmental fractures (n=2). Of these fractures, 10 were Gustilo Type II, five were Gustilo Type IIIA (two had delayed primary closure and three had split thickness skin grafting), and four were Gustilo Type IIIB (all had rotational flaps). Soft tissue injuries were classified according to criteria of Gustilo *et al*.,^2^,^18^,^19^ as modified by Chapman *et al*.^20^ In general, Type II injuries had a simple fracture pattern (transverse, oblique, spiral) associated with a wound larger than 1 cm but smaller than 10 cm. Type III fractures had open wounds larger than ten 10 cm or fracture patterns with segmental comminution. Within this category, Type IIIA fractures were those that could be closed, either by delayed primary closure or split-thickness skin graft, implying adequate soft tissue coverage of bone. Type IIIB fractures were those identified after initial debridement and stabilization which required rotational flap for soft tissue coverage [Table 1].

Nine patients had significant associated injuries including severe closed head injury in two patients, significant skin loss overlying large area around the fracture site in one patient, ipsilateral femoral shaft fracture in two patients (one had tibial bone loss of 8 cm (case 11) and the other was skeletally immature (case 16)), ipsilateral multiple metatarsal shaft fractures in two patients, and pulmonary contusion in two patients. All patients were treated using TSF using the following technique.

## Surgical procedure

At surgery a flat-top radiolucent table was used (OSI table, Orthopedic Systems, Union City, CA). The injured limb was prepared and draped as usual. All patients underwent emergent (within 8 h of admission) irrigation and debridement in the operating room with concomitant skeletal stabilization. Single surgeon was involved in the study. Cephalosporin antibiotics were administered perioperatively for all patients and gentamycin was added for Grade III cases for an average of 48 h, which could be continued for longer if deemed necessary by our infectious disease team and no barn yard injuries were encountered.^21^,^22^ No wounds were closed primarily. Twelve patients had delayed primary closure, three had split-thickness skin grafting, and four had rotational flap. All were performed between 3 and 10 days after injury at the discretion of the treating surgeon [Table 1]. Preplanned bone grafts were performed in a delayed fashion in cases 12 and 19.

Manual traction (by a surgical assistant pulling on the foot) was used to realign the bone ends and restore some length. The “rings-first” operative method was used in all cases. The diameter of the proximal and distal ring was selected in relation to the size of the patient's leg, with appropriate clearance from the skin to allow for additional swelling and local care of the soft tissue envelope. The diameter of the rings can be different to provide for a more contoured fixator. Next, a proximal or distal reference fragment was selected. The reference fragment was designed to be stationary in space with the opposite moving fragment rotating around it. The proximal and distal rings were attached to each bone segment independently.

**Table 1:** Clinical details of the patients

Case number	Age (years)	Gustilo‘s classification	Mechanism of injury	Associated injury	Fracture location	Coverage procedures	Cases requiring fasciotomy	Latency (months)	Time to healing (weeks)	Followup (years)	Final leg length discrepancy (mm)	Supplementary procedures
1	23	IIIB	MVA	Pulmonary contusion	Proximal/middle 1/3 junction	Rotational flap	No	0	30	5	4	No
2	28	II	All-terrain	Metatarsal fracture	Middle/distal 1/3 junction	Primary closure	No	0	24	2	0	No
3	19	II	Car v/s pedestrian	No	Distal 1/3	Primary closure	No	0	23	3	0	No
4	34	II	MVA	No	Middle/distal 1/3 junction	Primary closure	No	0	21	4	2	No
5	20	II	MVA	Metatarsal fracture	Middle/distal 1/3 junction	Primary closure	No	0	24	5	0	No
6	24	IIIB	MVA	Skin loss	Distal 1/3	Rotational flap	No	3	23	2	3	No
7	40	II	MVA	Pulmonary contusion	Middle/distal 1/3 junction	Primary closure	No	0	19	2	0	No
8	38	IIIA	MVA	No	Proximal 1/3	Split thickness skin graft	No	2.5	23	4	4	No
9	33	IIIA	MVA	Femur fracture	Middle 1/3 5 cm bone loss	Split thickness skin graft	No	3	44	5	2	Docking site iliac crest grafting
10	6	II	Car v/s pedestrian	No	Middle 1/3	Primary closure	No	0	9	4	0	No
11	45	II	MVA	No	Middle/distal 1/3 junction	Primary closure	Yes	0	21	3	0	No
12	28	IIIB	MVA	Head injury	Segmental	Rotational flap	No	0	24	5	5	Prophylactic iliac crest grafting
13	29	IIIA	MVA	No	Distal 1/3	Split thickness skin graft	No	2	22	4	3	No
14	24	IIIB	All-terrain	No	Middle/distal junction 6 cm bone loss	Rotational flap	No	3	46	3	3	Docking site iliac crest grafting
15	33	II	MVA	No	Middle 1/3	Primary closure	No	0	19	5	0	No
16	9	II	Car v/s pedisterian	Femur fracture		Primary closure	No	0	17	2	0	No
17	18	II	Car v/s pedisterian	No	Distal 1/3	Primary closure	No	0	19	2	2	No
18	29	IIIA	MVA	No	Proximal/middle 1/3 junction	Split thickness skin graft	No	0	22	3	3	No
19	18	IIIA	MVA	Head injury	Segmental	Split thickness skin graft	Yes	0	20	4	5	Prophylactic iliac crest grafting

**Figure 1:**
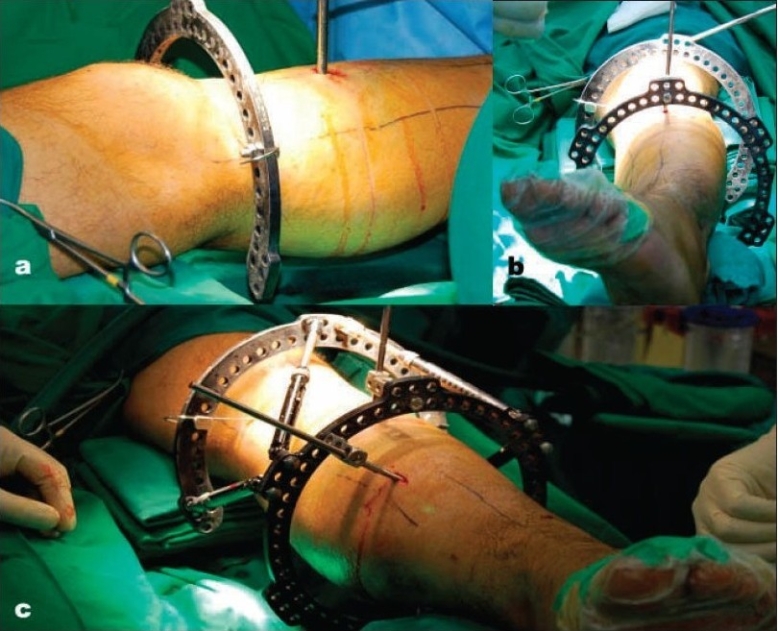
Clinical photographs showing (a) Patient leg after mounting of the first ring on a tensioned Olive wire. (b) Patient leg after mounting of the second ring on half pin. (c) Frame assembled after traction with the six TSF struts connecting first and second ring.

The rings were placed in an orthogonal manner, so that each ring is perpendicular to the long axis of its segment. A transverse 1.8-mm smooth transosseous wire or 6-mm half-pin was next inserted in the reference fragment [Figure 1a]. The wire should be perpendicular to the bone axis or parallel to the adjacent joint. The fixator ring was mounted to this reference wire or screw. At least three 6-mm hydroxyapatite-coated external screws (Orthofix, Richardson, TX) were used in the diaphysis on each side of the fracture. In the metaphyseal segment 3 or 4, 1.8-mm transosseous wires were placed in the frontal plane and 1 or 2 external screws in the sagittal plane. After the rings were fixed to the bone [Figure 1b], six struts were attached to the nonparallel rings, and the strut length was determined by the ring position [Figure 1c]. Best possible reduction was achieved by manipulating TSF standard strut length, and in the last 10 cases, the fast fix struts that make changing the strut length easier and faster, dressing was then applied. In case of bone loss of more than 3 cm, a frame that allows bone transport was applied (cases 9 and 14).

Anteroposterior and lateral radiographs were then obtained. The radiographs were analyzed for the deformities present. The total residual online program (Smith and Nephew Richards, Memphis, TN) was used in all cases. To use the previously mentioned computer program, the anatomic side involved and 13 parameters that describe the fixator itself, the deformity between the bone ends, and the position of the fixator to the bone were entered into the computer. The deformity parameters include the 6 planes of deformity.

There may be three angulations and three translations in the frontal, sagittal, and axial planes. These were measured in degrees and millimeters from the anteroposterior (AP) and lateral radiographs. Before the translation of the bone ends can be measured, the surgeon selects a point on the reference fragment (stationary fragment), which was termed the origin [a bone edge (cortex or spike) was marked on the AP and lateral radiographs]. On the moving fragment, a second point was chosen, either on the same cortex or in the depression of the spike. This was termed the corresponding point and, when repositioned to the origin, the bone ends were reduced anatomically. The translation was calculated as the distance between the origin and corresponding point in the three planes previously mentioned. The final data that were entered by the operator were the mounting parameters. The distance between the center of the reference fragment ring and the origin was determined using the AP radiograph. This represents the AP frame offset. The distance between the frame center and the origin on the lateral radiograph was the lateral frame offset. The axial frame offset was the longitudinal distance from the reference ring to the origin measured from the AP radiograph. A rotary frame offset was added according to the frame position on the leg, the number of degrees and the direction were entered in the rotary frame offset box, and the computer through the total residual online program generates the length to which each strut is adjusted to gain perfect reduction. These adjustments were easy to make and were done gradually in the early postoperative period to avoid excessive soft tissue damage, and if anatomic reduction was not present on the postreduction radiographs, fine tuning using the total residual mode again was then used over the next few days to obtain perfect alignment, according to the adjustment schedule, which was generated by the computer program.

Two patients who had compartment syndrome underwent four compartment fasciotomy with delayed skin grafting (cases 11 and 19). Typically, for an isolated noncomminuted tibial fracture, partial weight-bearing was initiated on the first postoperative day and gradually advanced to full weight-bearing with the help of crutches within 4-6 weeks. Fractures were labelled as healed after evidence of callus in three cortices in AP and lateral X-rays and lack of tenderness at the fracture site. Wire and pin tract infection was classified according to the Dahl classification as Grade 1 inflamed requiring daily pin care only, Grade 2 serous discharge requiring oral antibiotics, Grade 3 purulent discharge requiring oral antibiotics, Grade 4 osteolysis requiring pin removal, and Grade 5 ring sequestrum requiring debridement.^23^ TSF was removed in an outpatient surgery unit under sedation, and no postoperative immobilization was needed in all cases. Patients were then seen after 1 week for pin site wound check, and then every 3 months for two visits then yearly.

## RESULTS

**Figure 2:**
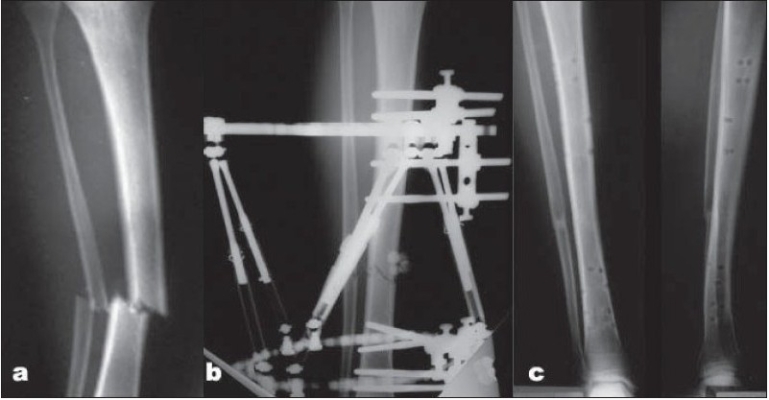
(a) Radiograph showing tibial shaft fracture in a 20 years old male patient (case 5). (b) Anteroposterior radiograph immediately after application of TSF with reduction of the fracture (c) Anteroposterior and lateral radiographs 6 months after removal of fixator showing radiological union.

**Figure 3:**
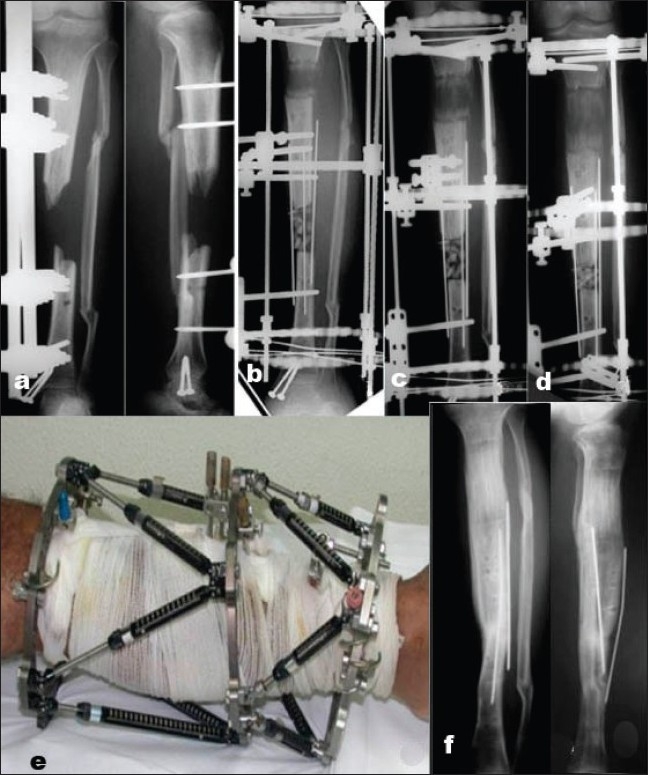
(a) Anteroposterior radiographs of open tibial shaft fracture with bone loss (case 14). (b,c,d) Anteroposterior radiographs after application of the TSF and progress of bone transport. (e) Clinical picture with TSF rings and Struts. (f) Anteroposterior and lateral radiographs 3 months after fixator removal with consolidated bone transport, healed docking site, and anatomic alignment is present.

Seventeen fractures healed over a mean of 25 weeks (range, 9-46 weeks). Patients remained in the external fixator for a mean of 27 weeks (range, 11-49 weeks), which varied from healing time because most of the patients waited for the nearest time off and the availability of a slot in the outpatient surgical care unit [Figure 2 a-c]. Two patients had bone transport (cases 9 and 14); the first was of 5 cm and the second was for 6 cm as a result of bone loss at the time of the open fracture. Both had bone grafting of the docking site and had the frame on for 44 and 46 weeks, respectively [Figure 3]. The TSF strut adjustment allowed for correction of the common procurvatum valgus deformity at the lengthening site and the malalignment at the docking site.

All patients were able to perform activities of daily living with no difficulty and involved in sports when last seen including patients with other significant associated injuries. Seventeen patients were not experiencing any pain in their last follow-up; one patient with significant skin loss had minimal pain required occasional use of nonsteroidal anti-inflammatory drugs, and the other continued to have mild pain at the rotational flap site that did not required any analgesics. No patient was noted to be more than 5° malrotated on clinical examination. No patient had a functional leg length discrepancy. There were no intraoperative complications. Postoperative complications included pin tract infection in 12 patients, and those who had Grade 2 or 3 infection resolved with oral antibiotics, no patient developed osteolysis or ring sequestrum, neurovascular injury, or refracture. Seventeen patients had a normal range of motion of their knee and ankle on clinical examination during their last follow-up visit, and the remaining two had limitation of their ankle range of motion from 5° of dorsiflexion to 10° of plantar flexion as a result of significant skin loss around fracture site in one and a segmental fracture in the other.

**Figure 4:**
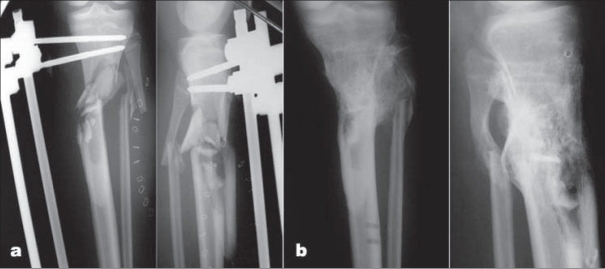
(a) Anteroposterior (AP) and lateral radiographs of (case 19) showing comminuted tibial shaft fracture in an 18 years old male patient. (b) AP and lateral radiographs 6 months after removal of fixator showing radiological union.

All the fractures ultimately united including two patients (case 12 and 19) with severely comminuted fracture who had a preplanned autogenous iliac crest bone grafting 2 months after the initial injury while still in the external fixator and both fractures healed uneventfully [Figure 4] after (24 weeks and 20 weeks respectively). There were no loss of reduction or return to the operating room for remanipulation and no patient developed a malunion. The mean preoperative malalignment on the anteroposterior radiograph of the tibia was 16° (range, 5-35°). The mean preoperative displacement on the anteroposterior radiograph of the tibia was 62% (range, 40-100%). The mean preoperative malalignment on the lateral radiograph of the tibia was 10° (range, 0-15°). The mean preoperative displacement on the lateral radiograph of the tibia was 60% (range, 5-100%). The mean final malalignment on the anteroposterior radiograph of the tibia was 2° (range, 0-4°). The mean final displacement on the anteroposterior radiograph of the tibia was 3% (range, 0-6%). The mean final malalignment on the lateral radiograph of the tibia was 3° (range, 0-5°). The mean final displacement on the lateral radiograph of the tibia was 2% (range, 0-5%).

## DISCUSSION

External fixation has a clear role in the management of open tibial fractures, specifically high-energy injuries with extensive soft tissue damage. The use of an external fixator for the open tibial fracture is well described in the literature.^1^,^5^,^6^,^24^–^30^ Bone transport with circular external fixator has been shown to be a reliable method to treat segmental bone loss with success reported between 75% an 100% success.^6^,^8^,^31^,^32^ however a number of studies reported significant complications such as infection, delayed union, refracture, limb overgrowth, malunion, need for remanipulation, and joint stiffness.^24^–^29^ Complications related to pin site problems have been well described in tibial external fixation.^33^,^34^ Henley *et al*. concluded that unreamed interlocking intramedullary nails are more efficacious than half-pin external fixators in the treatment of Type II, IIIA, IIIB open fractures of the tibia, in particular with regard to maintenance of limb alignment,1 Ideally fracture care should be as minimally invasive as possible and avoiding the presence of foreign material such as intramedullary nails. Internal fixation has the best chance at anatomic reduction, but it is also the most invasive form of treatment and carries a higher risk of infection. Intramedullary nails works best in diaphyseal transverse fracture patterns, but unstable oblique, spiral, and comminuted fractures present a greater challenge for intramedullary nails.^35^,^36^ Circular external fixators provide a stable construct for the varying forms of tibial fractures.^5^ Advanced techniques using the Ilizarov method may provide healing of the associated large soft tissue defects without the need for flap through acute shortening(docking) at fracture site to facilitate wound closure.^6^ In comparing external fixation to undreamed locking intramedullary nailing (IMN) of the open tibia, Tornetta *et al*. reported on 29 Type IIIB open tibial fractures (15 treated by IMN and 14 with external), although the differences in healing and range of motion were not statistically significant the conclusion was made that locked nonreamed nailing is the treatment of choice for Grade IIIb open tibial fractures.^37^

The current study discusses a group of high-energy open tibial shaft fractures; this high-energy trauma caused extensive soft tissue damage. Here, the author used the TSF, which is a modern multiplanar external fixator that combines the ease of application plus computer accuracy in the reduction of tibial fractures through inputting 13 parameters into a computer software program that provides the proper adjustments needed to reduce the fracture. Indirect reduction of the fracture is achieved by realigning one fracture end to the other through a spatial point of rotation.^5^ This method of treatment allows immediate stabilization of the fracture, facilitates access to soft tissues for reconstruction, and obviates the need for immobilization. Basically, the TSF represents the ultimate form of indirect reduction techniques, which is will documented through the corrections achieved. In this study, no significant postoperative complications occurred, only 12 patients had pin tract infection and all resolved with oral antibiotics; no patient developed osteomyelitis, neurovascular injury, refracture, delayed union, limb overgrowth, malunion, need for remanipulation, and significant joint stiffness.

Our wire and pin site infections matches that in the literature. Dahl *et al*. stated that nearly all patients with ring fixator experience pin or wire infection (grade 1-3); however, osteolysis or ring sequestrum occur only in 5% of cases.^23^

Our study compares favorably with other published reports in the literature; particularly those that involved the use of the TSF in the tibia include a report by Biniski with 50 acute tibial fractures with only 12 open tibial fractures treated with the TSF. A 93% union rate occurred, with only one refracture (2%) and two nonunions (4%). The three failures were treated successfully with a second procedure, and anatomic alignment was achieved in 96% of patients.^5^ The second report was the author's work on pediatric and adolescent tibia shaft fractures that included 10 tibia fractures; all patients were boys with an average age of 12 years (range, 8-15 years). Mean duration of follow-up was 3.1 years. These include five open fractures three of which were included in this study. All fractures healed over a mean of 18 weeks. Postoperative complications included pin tract infection in five patients and concluded that TSF is an effective definitive method of tibial fracture care with the advantage of early mobilization and ability to postoperatively manipulate fracture into excellent alignment.^38^

This study has limitations. It is a retrospective study and includes a small number of severe injuries specifically Type IIIB patients. Functional data were limited. Despite these limitations, this report discusses a peculiar group of patients that are difficult to treat, and who were managed with a relatively new fixator (the TSF) with a promising end result. In summary, the TSF is an external fixator for definitive open tibia fracture care in the pediatric, adolescent, and adult patients from reduction to healing. The technique is minimally invasive to the fracture site. The fracture site can be manipulated postoperatively over few days into excellent alignment in outpatient basis without any need for remanipulation in the operating room, and is stable enough to allow early functional weight-bearing and active motion of adjacent joints as demonstrated in this study.
